# Cryptococcal Infection in a Patient With Chronic Lymphocytic Leukaemia Receiving Acalabrutinib, a Bruton’s Tyrosine Kinase Inhibitor: A Case Report

**DOI:** 10.7759/cureus.99241

**Published:** 2025-12-14

**Authors:** Abdul-Razaq N Al-Shaker, Ansh Agarwal, Muhammad Talha Saeed, Zena Marney

**Affiliations:** 1 Medicine, Hywel Dda University Health Board, Carmarthen, GBR

**Keywords:** bruton’s tyrosine kinase, chronic lymphocytic leukemia (cll), cll treatment, cryptococcosis. disseminated cryptococcosis, tyrosine kinase receptor inhibitors

## Abstract

A male patient in his mid-sixties with chronic lymphocytic leukaemia (CLL), Binet stage B, receiving the Bruton’s tyrosine kinase (BtK) inhibitor acalabrutinib, presented with fever and other non-specific systemic symptoms. Following the investigation, they were found to have Cryptococcal meningitis. Cryptococcal infections are known to occur in immunocompromised patients, but they are rarely encountered or thought about during the initial acute medical assessment. This case highlights the importance of considering the possibility of opportunistic infections and the atypical presentation of such infections in immunocompromised patients, including those on BtK inhibitors.

## Introduction

Chronic lymphocytic leukaemia (CLL) is the most common form of leukaemia and occurs more commonly with advancing age [1]. Bruton’s tyrosine kinase (BtK) inhibitors such as acalabrutinib have revolutionised CLL management. However, these agents impair B-cell receptor signalling and alter immune responses, potentially predisposing patients to infections [2]. 

We present a case of cryptococcal infection in a patient with CLL treated with acalabrutinib, highlighting the need for vigilance for opportunistic infections and timely investigation in immunocompromised individuals receiving targeted therapies.

## Case presentation

A male patient in his mid-sixties with CLL, Binet stage B, who had been started on acalabrutinib nine months before admission, presented with non-specific symptoms including fever, lethargy, increased night sweats and general malaise.

Initially, blood tests showed mild neutropenia and an elevated C-reactive protein (CRP) of 100 mg/L. During the investigation, cultures were obtained, and he was commenced on broad-spectrum antibiotics for febrile neutropenia; acalabrutinib was withheld. He was monitored closely and isolated in a cubicle; blood cultures subsequently returned positive for Cryptococcus.

Following discussion with a microbiologist, the patient was commenced on amphotericin B and flucytosine, and concern was raised regarding the possibility of cryptococcal meningitis despite the absence of meningism on clinical assessment. As a result, a lumbar puncture was performed, and CSF was sent for culture. The opening pressure was 11 cm H₂O, and the CSF cryptococcal antigen returned positive (Table 1).

**Table 1:** First lumbar puncture (LP)/cerebrospinal fluid (CSF) sample.

RBCs	WBC	Protein	Glucose (CSF)	Stain	Culture and virology	Cryptococcal antigen
0 x 10^6/L	53 x 10^6/L (Polymorph: 10%, lymphocyte: 90%)	0.72 g/L (0.15-0.45)	3.2 mmol/L	On Gram stain, white blood cells were seen, but no organisms were identified. On the India ink stain, no encapsulated yeasts were seen.	No growth in culture and negative virology	Detected

The patient showed significant improvement with antifungal treatment, became afebrile, and all other systemic symptoms resolved. A second CSF sample was obtained after three weeks of antifungal therapy, and results demonstrated ongoing lymphocytosis and a positive cryptococcal antigen test despite clinical improvement (Table 2).

**Table 2:** Second lumbar puncture (LP)/cerebrospinal fluid (CSF) sample (after three weeks of antifungals).

RBCs	WBC	Protein	Glucose (CSF)	Stain	Culture and virology	Cryptococcal antigen
3,600 x 10^6/L	27 x 10^6/L (polymorph: 10%, lymphocyte: 90%)	0.92 g/L (0.15-0.45)	2.8 mmol/L	On Gram stain, WBCs were seen, but no organisms were seen. On the India Ink stain, no encapsulated yeasts were seen.	No growth in culture and negative virology	Detected

Following discussion with a microbiologist, the patient was felt well enough to be discharged on high-dose fluconazole, with regular outpatient follow-up, especially in view of improving CRP (Table 3).

**Table 3:** Blood test results.

Investigations	Results on admission	Discharge results	Unit of measurement	Reference range
Haemoglobin	129	115	g/L	130-180
White cell count	4.4	25.4	x10^9/L	4.0-11.0
Platelet count	114	172	x10^9/L	150-400
Haematocrit	0.41	0.37	L/L	0.40-0.52
Mean cell volume	83	83	fL	80-100
Red cell count	4.93	4.43	x10^12/L	4.50-6.00
Mean cell haemoglobin	26.2	25.9	pg	27.0-33.0
Red cell distribution width	15.4	17.6	%	11.0-14.8
Neutrophils	2.7	3.5	x10^9/L	1.7-7.5
Lymphocytes	1.4	19.0	x10^9/L	1.0-4.5
Monocytes	0.1	0.3	x10^9/L	0.2-0.8
Eosinophils	0.0	0.1	x10^9/L	0.0-0.4
Basophil	0.0	0.3	x10^9/L	0.0-0.1
Serum C-reactive protein	100	5	mg/L	<5
Serum sodium	130	139	mmol/L	133-146
Serum potassium	3.9	4.3	mmol/L	3.5-5.3
Serum urea	13.6	6.7	mmol/L	2.5-7.8
Serum creatinine	104	96	mmol/L	58-110
Serum bilirubin	17		umol/L	<21
Serum alkaline phosphatase	54		U/L	30-130
Serum alanine transaminase	11		U/L	<41
Serum albumin	48		g/L	35-50
Serum calcium (adjusted)	2.28		mmol/L	2.20-2.60
Serum magnesium	0.87		mmol/L	0.70-1.00
Serum total protein	76		g/L	60-80

From a CLL perspective, following cessation of acalabrutinib, the patient became progressively anaemic, with a rising lymphocyte count (Table 3). He was therefore considered for alternative immunosuppressive treatment.

## Discussion

Chronic lymphocytic leukaemia

CLL is the most common form of leukaemia and occurs more commonly with advancing age. Patients may be asymptomatic at the time of diagnosis, and as such diagnosis can be incidental when checking routine blood tests. Its prognosis depends upon multiple factors, including the clinical stage of the disease at the time of diagnosis, the patient’s age and the pace of disease progression. Patients with CLL are more prone to infections due to functional neutropenia and immune failure due to reduced immunoglobulins [1].

Fungal infections are not typically observed in CLL in the absence of treatment with corticosteroids or other immunosuppressive therapy for autoimmune complications. Cryptococcal meningitis, pneumonia and fungaemia are well-recognised occurrences in patients with CLL and are associated with significant morbidity and mortality [3].

CLL treatment depends upon the stage of the disease at the time of diagnosis and is often treated expectantly in the early stages and immediately in the later stages. Treatment modalities include steroids and chemotherapy. Erythropoietin can be considered to avoid the need for transfusions. Immunoglobulin replacement and prophylactic antimicrobial treatment should also be considered [1].

More specific treatments such as chemotherapies and targeted immune therapies are considered according to the patient’s genetic profile, comorbidities, functional status and patient’s preferences. Examples of such treatments include BtK inhibitors such as acalabrutinib and ibrutinib, anti-CD20 antibodies such as obinutuzumab and rituximab, and purine inhibitors (fludarabine) given together with cyclophosphamide and rituximab [4].

Tyrosine kinase and BtK and its inhibitors

Tyrosine kinase receptors (TKRs) are transmembrane immunoglobulin-like molecules; a mutation in this receptor can lead to accelerated cellular proliferation, extended cell survival, increased angiogenesis and cellular migration. Tyrosine kinase inhibitors (TKIs) are a group of medications that inhibit tyrosine kinase receptors (TKRs), leading to suppression of tumour growth and spread [5].

BtK is a non-receptor kinase that plays a crucial role in oncogenic signalling that is critical for the proliferation and survival of leukaemic cells in many B-cell malignancies. The orally administered irreversible BtK inhibitor ibrutinib is associated with high response rates [2].

Acalabrutinib is a second-generation BtK inhibitor with minimal off-target effects compared with ibrutinib, thereby limiting adverse events. Ongoing trials in high-risk relapsed CLL are comparing acalabrutinib with ibrutinib [3].

As acalabrutinib is a relatively new agent in the management of CLL, there are limited published case reports describing cryptococcal infection in patients receiving this treatment. We therefore believe this case report is valuable in highlighting the atypical presentation of this opportunistic infection.

## Conclusions

Targeted therapies, such as BTK inhibitors, have revolutionised the management of CLL. Acalabrutinib is a second-generation BTK inhibitor believed to have a more favourable adverse event profile compared with ibrutinib. This case describes a patient with CLL who presented with fever and non-specific symptoms. Investigations revealed cryptococcal meningitis, which was successfully treated with a prolonged course of antifungal therapy. This highlights the importance of considering opportunistic infections in this patient group, as well as the potential for atypical presentations.
